# Assessment of Third Molar-related Symptoms Affecting Quality of Life using Nepali Version of Oral Health Impact Profile-14: A Descriptive Cross-sectional Study

**DOI:** 10.31729/jnma.8546

**Published:** 2024-04-30

**Authors:** Bikash Chaudhary, Sujaya Gupta, Yashuma Tiwari, Sangya Mukhiya, Dikshya Silwal, Shristi Shakya

**Affiliations:** 1 Department of Oral and Maxillofacial Surgery, Kathmandu Medical College Teaching Hospital, Duwakot, Bhaktapur, Nepal; 2 Department of Periodontics and Oral Implantology, Kathmandu Medical College Teaching Hospital, Duwakot, Bhaktapur, Nepal; 3 Kathmandu Medical College Teaching Hospital, Duwakot, Bhaktapur, Nepal

**Keywords:** *impacted tooth*, *oral health*, *quality of life*, *third molar*, *tooth extraction*

## Abstract

**Introduction:**

Impacted third molars often cause pain, infections, swelling, and functional limitations. This study is an attempt to assess impacted third molars-related symptoms affecting quality of life using standardised Nepali version of oral health impact profile-14 (OHIP-14) questionnaire.

**Methods:**

This descriptive cross-sectional study was conducted at Kathmandu Medical College Teaching Hospital from October 2021 to February 2022 after institutional ethical approval. Patients with impacted third molars were included by convenience sampling technique. Patients with psychiatric illness, taking psychotropic drugs, pregnant, and lactating females were excluded. third molars-related symptoms were recorded in OHIP-14 questionnaire. Data entered in Microsoft Excel sheet were analysed. The findings have been presented as frequency, percent, mean, and standard deviation. The point estimate was calculated at a 95% Confidence Interval.

**Results:**

Mean OHIP-14 score of participants was 21.77±11.59. Due to TM, "pain in the mouth" had score of (2.33±1.24) and followed by "uncomfortable experience on eating food" (2.12±2.15). Among seven OHIP-14 dimensions, "physical pain" with two items OHIP3 and OHIP4 had score of 4.53±2.19 implying most participants had "quite a lot" of physical pain due to TM: OHIP3 = 194 (50.2%) and OHIP4 = 183 (47.3%).

**Conclusions:**

Impacted third molars-related symptoms were affecting quality of life of participants.

## INTRODUCTION

World Health Organization (WHO) defines Quality of Life (QoL) as individuals' perception of their position in life in context of culture and value systems in which they live and in relation to their goals, expectations, standards, and concerns.^1^ Impacted third molars (TM) often manifest as pericoronitis resulting in pain, infections, swelling, and functional limitations that affect oral health-related quality of life.^2-4^

Decision-making regarding teeth extraction is vital aspect impacting individual's quality of life.^2,4,5^ The Nepali version of oral health impact profile-14 (OHIP-14) is a standardised tool that can be used to assess TM symptoms relating to oral health-related QoL.^6^ Studies have highlighted need for specific research investigating retention versus extraction of asymptomatic TMs and their impact on QoL in short and long term.^3,5-8^

Thus, this study is an attempt to assess the symptoms related with impacted third molar using a standard tool, the Nepali version of OHIP-14 questionnaire.

## METHODS

This descriptive cross-sectional study was conducted among 387 individuals who visited the dental departments of Kathmandu Medical College and its Teaching Hospitals (KMCTH) at Duwakot, Bhaktapur and at Sinamangal, Kathmandu, Nepal from October 2021 to February 2022. Ethical approval was obtained from the Institutional Review Committee of the same institute (Reference number: 110202102) and informed consent was taken from all the study participants prior to the study. Individuals with problems related to third molar, individuals who had undergone extraction of third molar, and individuals willing to participate were included in the study. The exclusion criteria were individuals unwilling to participate in the study, individuals with psychiatric illnesses or those taking medications that affect their mental state (such as antidepressants and benzodiazepines), and pregnant ladies and lactating mothers. For calculation of sample size it was hypothesised that prevalence of physical, psychological and social disability each will be 50% affecting the quality of life. The sample size was calculated using the following formula:


$$\begin{array}{l} n={\text{Z}}^{2}\times \frac{\text{p}\times \text{q}}{{\text{e}}^{\text{2}}}\\ \;=\;383.29\approx 384 \end{array}$$

Where,

Z = 1.96 at 95% Confidence Interval (CI)
p = prevalence of third molar impaction, 47.63%^7^
e = 0.05 (5% margin of error)

Individuals visiting the study site who fulfilled the study criteria participated in the study after their approval. A set of questionnaire consisted of: 1. Informed consent; 2. Demographic data; 3. Questionnaire to assess third molar-related symptoms affecting quality of life. It was measured using the standardized Nepali version of OHIP-14.6 The OHIP-14 questionnaire includes seven dimensions with 14 items. The seven dimensions include: functional limitations (OHIP1 and OHIP2), physical pain (OHIP3 and OHIP4), psychological discomfort (OHIP5 and OHIP6), physical disability (OHIP7 and OHIP8), psychological disability (OHIP9 and OHIP10), social disability (OHIP11 and OHIP12), and handicap (OHIP13 and OHIP14). The higher the average value of seven dimensions, the more negative impact of the thirdmolar on quality of life can be considered. The response to the questionnaire can be recorded as: "none at all (0)", "seldom (1)", "very little (2)", "pretty much (3)", and "quite a lot (4)". In the initial screening, none of the participants responded "seldom" to any item. Thus, for simplicity, the authors have analysed only three responses in this study. The two responses "none at all" and "seldom" were combined and the responses "pretty much" and "quite a lot" were combined. The data were entered in Microsoft Excel sheet and analysed. The findings are presented as frequency, percent, mean, and standard deviation. The point estimate was calculated at a 95% CI.

## RESULTS

The symptoms related to impacted third molar from 387 individuals seeking dental extraction, were recorded on OHIP-14 questionnaire and analysed. The age of the participants was 29.85±11.84 years (median and mode = 25 years; standard error of mean = 0.61). Majority of the participants were female (215, 55.56%) and 172 (44.44%) were male. The participant reported 'quite a lot" in physical disability as unsatisfactory diet 106 (27.39%), interruption of meal 91 (23.50%); in psychological disability as difficult to relax 118 (30.49%), feeling embarrassed 46 (11.89%); in social disability as irritable with others 85 (21.96%), difficulty doing usual jobs 79 (20.42%) (Table 1).

**Table 1:** Response to the Nepali version of oral health impact profile-14 questionnaire (n = 387).

Impact of third molar-related symptoms	None at all, n (%)	Very little, n (%)	Quite a lot, n (%)	Mean±S.D.
**Functional limitation:**				2.57±2.42
OHIP1. Difficulty in pronouncing words	187 (48.32)	105 (27.13)	95 (24.55)	1.34±1.38
OHIP2. Taste has worsened	195 (50.39)	114 (29.46)	78 (20.16)	1.23±1.32
**Physical pain:**				4.53±2.19
OHIP3. Pain in mouth	64 (16.54)	129 (33.33)	194 (50.13)	2.33±1.24
OHIP4. Uncomfortable eating food	77 (19.90)	127 (32.82)	183 (47.28)	2.12±2.15
**Psychological discomfort:**				3.98±2.18
OHIP5. Self-consciousness	97 (25.06)	128 (33.08)	162 (41.86)	1.98±1.27
OHIP6. Tense feeling	88 (22.74)	148 (38.24)	151 (39.02)	2.01±1.24
**Physical disability:**				3.10±2.28
OHIP7. Unsatisfactory diet	140 (36.18)	141 (36.43)	106 (27.39)	1.6±1.30
OHIP8. Interruption of meals	149 (38.50)	147 (38)	91 (23.50)	1.51±1.29
**Psychological disability:**				2.58±2.12
OHIP9. Difficult to relax	136 (35.14)	133 (34.37)	118 (30.49)	1.66±1.34
OHIPIO. Feeling embarrassed	236 (60.98)	105 (27.13)	46(11.89)	0.92±1.20
**Social disability:**				2.55±2.32
OHIP11. Irritable with others	198 (51.17)	104 (26.87)	85 (21.96)	1.21±1.31
OHIP12. Difficulty doing usual jobs	173 (44.70)	135 (34.88)	79 (20.42)	1.34±1.28
**Handicap:**				2.44±2.32
OHIP13. Life less satisfying	182 (47.03)	136 (35.14)	69 (17.83)	1.34±2.04
OHIP14. Totally unable to function	194 (50.13)	131 (33.85)	62 (16.02)	1.18±1.26
**Total OHIP-14 score**				21.77±11.59

The overall mean score of OHIP-14 questionnaire among the study population was 21.77±11.59 (Table 1). The mean score of the item "pain in the mouth "due to third molar problem was most significant which was 2.33±1.24 followed by "uncomfortable experience on eating food" which was 2.12±2.15. The mean score of dimension physical pain with two items (OHIP3 and OHIP4) was 4.53±2.19 which means the majority of individuals had "very little" to "quite a lot" of physical pain due to third molar. The females had slightly lower mean scores (21.33±11.42) than male participants(22.32±11.82).

## DISCUSSION

In this study, 387 individuals were included prospectively,who participated and responded to OHIP-14 questionnaire as they reported to the dental department. The responses when analysed showed that the impact of pain in mouth and discomfort due to third molar-related symptoms was seen in the highest number of the participants. Half of the individuals, i.e. 194 (50.12%) were found to have "pain in the mouth" and 183 (47.28%) had "uncomfortable feeling while eating food" and responded as "quite a lot" to the questionnaires. Few people 62 (16.02%) responded as "quite a lot" to the question "totally unable to function because of third molar-related problems". In present study, the highest mean value correlated to the dimension "physical pain" with a mean value of 4.53± 2.19 which is in coherence with the finding of the study conducted in Jerusalem, Israel where the mean value was 4.2 ± 2.0.^8^ This implies that the participants in both studies suffered physical pain due to impacted third molars to a degree that affected their quality of lives. This can be easily prevented, if individuals visited their dentists at least once a year if not the recommended twice. That way, any impacted third molars will be dealt with in an appropriate manner before they start affecting the daily living of the individuals.

The preoperative symptoms related to impacted third molars that may affect oral health-related quality of life in participating individuals was assessed. Oral health-related quality of life measures oral health by influencing physical and social well-being.^9^ High OHIP-14 score reflects negative physical, psychological, and social impact. In this study, the authors assessed the preoperative OHIP using Slade et al. questionnaire translated in Nepali by Vikram and Singh.^6,10^ The OHIP-14 questionnaire was developed to be more global, covering a wide variety of oral health conditions and treatments including third molars.^10^ The OHIP-14 questionnaire was translated to Nepali version and standardised.^6^ The specific conditions, such as psychological discomfort, psychological disability, and social disability, havepreviously shown shortterm effects of third molar surgery.^11^ Individuals seeking third molar surgery due to symptoms have a higher likelihood of experiencing adverse impacts on their quality of life as compared to asymptomatic individuals. ^5^

In the current study 95 (24.55%) individuals responded to "ever felt difficulty in pronouncing any words" as "quite a lot" with a mean value of 1.34±1.38. This finding was in contrast to the study conducted in Makassar, Indonesia where 41 (20.3%) responded as "often" and 14 (6.9%) responded as "always" to the same questionnaire with a mean value of 1.79±0.99.^12^ In current study, 78 (20.16%) responded to the questionnaire "sense of taste has worsened" as 'quite a lot" in contrast to the study conducted in Karnataka, India where only 10 (4%)responded as "often/fairly often" which was related with age,history of loss of the tooth, and the reason behind the toothextraction.^2^

In the current study, 162 (41.86%) individuals responded to the questionnaire "self-consciousness" where the mean value was 1.98±1.27 and 151 (39.02%) individuals responded to the questionnaire "felt tense" with the mean value 2.01±1.24. The mean of these two items (OHIP5 and OHIP6) of the dimension "psychological discomfort" was 3.98±2. which was similar to the study conducted in Makassar,Indonesia with the mean value of 2.07.^12^

In this study, 118 (30.49%) responded as "quite a lot" to the questionnaire "difficult to relax" and only few people 46 (11.89%) responded as "quite a lot" to the questionnaire "feeling of embarrassment due to problems in the teeth". The response to a question "feeling of embarrassment due to problem related to third molar" had the least mean value 0.92±1.20 which means almost all the respondents answered none to very little. This finding was in contrast to study where the least mean value to the question "unable to function due to third molar" was 1.65.^12^

Most of the individuals were in the category of around 25 years of age (Median and Mode = 25 years) which was consistent with the previous study conducted in Karnataka, India.^2^ Majority of the individuals were female which was similar to previous studies conducted.^5,12^ This finding was in contrast to another study conducted where the majority of patients were male.^2^

The mean OHIP-14 score among the participants of this study was 21.77±11.59. The score was in line with another study conducted where the mean score was 22.5±8.3.^2^ The mean OHIP score was higher than other study conducted which was 7.1±8.0.^13^ The difference could be due to difference in type of population under investigation. The female participants had similar mean score (21.33±11.42) as male participants (22.32±11.82). This is similar to another study conducted in Nepal, where authors reported an insignificant difference in mean OHIP-14 score among males and females.^13^ However, the values are lot higher in current study. The difference can be attributed to the composition of population. The previous study was done in rural population with only 200 sample size whereas current study had urban population.

The research findings will be helpful to provide baseline information about symptoms related with impacted third molars and its probable effect on oral and general health and well-being. The findings may also help to provide the dental practitioners with more knowledge that could help when deciding whether or not to extract third molars. This study limits its finding to the proportion, a comparative arm to look into the quality of life would give more accurate results, therefore association of symptoms with third molar impaction can be planned to verify the findings of this study.

## CONCLUSIONS

The higher OHIP-14 score implies negative impact on quality of life. Results suggest impacted TM-related symptoms was affecting the quality of life of the study participants. Timely extraction of any impacted teeth can prevent most impaction related symptoms. Hence, it is recommended that for the prevalent age groups, timely radiographs be done to assess the status and need for extraction of the impacted third molars.
